# Functional septate junctions between cyst cells are required for survival of transit amplifying male germ cells expressing Bag of marbles

**DOI:** 10.1101/2024.04.02.587826

**Published:** 2024-04-03

**Authors:** Cameron W. Berry, Margaret T. Fuller

**Affiliations:** 1 Department of Developmental Biology, Stanford University School of Medicine, USA.; 2 Department of Genetics, Stanford University School of Medicine, USA.

**Keywords:** Septate Junctions, Somatic support cells, Germ cell survival, Spermatogenesis

## Abstract

In adult stem cell lineages, the cellular microenvironment plays essential roles to ensure the proper balance of self-renewal, differentiation and regulated elimination of differentiating cells. Although regulated death of progenitor cells undergoing proliferation or early differentiation is a feature of many tissues, mechanisms that initiate this pruning remain unexplored, particularly in the male germline, where up to 30% of the germline is eliminated before the meiotic divisions. We conducted a targeted screen to identify functional regulators required in somatic support cells for survival or differentiation at early steps in the male germ line stem cell lineage. Cell type-specific knockdown in cyst cells uncovered novel roles of genes in germline stem cell differentiation, including a previously unappreciated role of the Septate Junction (SJ) in preventing cell death of differentiating germline progenitors. Loss of the SJ in the somatic cyst cells resulted in elimination of transit-amplifying spermatogonia by the 8-cell stage. Germ cell death was spared in males mutant for the differentiation factor *bam* indicating that intact barriers surrounding transit amplifying progenitors are required to ensure germline survival once differentiation has initiated.

## Introduction

During animal development, cells arising from different embryonic origins come together to form functional organs, often delimited by one or more layers of cells arranged in an epithelium. Epithelial cells commonly have apical basal polarity and in many cases form occluding junctions with their neighbors in the layer, creating a barrier that seals off the internal cells of the organ from the rest of the body (Marchiando et al., 2010). Barriers are also formed by epithelial cells on smaller scales within tissues as in the mammalian testis, where proliferating progenitor cells derived from germline stem cells are separated from differentiating meiotic pachytene spermatocytes via a junctional barrier created by somatic Sertoli cells (Mruk & Cheng, 2015).

Occluding junctions contain bands of transmembrane cell-adhesion proteins that bind in the extracellular space to partners on neighboring cells, forming a barrier that prevents diffusion of molecules above a certain size between adjacent cells. Vertebrate occluding junctions include tight junctions (TJs) in epithelial cells and septate junctions (SJs) found in glial sheets, while most insects only have SJs (Tepass et al., 2001). At least 20 components of the SJ complex are known in *Drosophila*, including the transmembrane components Neurexin-IV (*Nrx-IV*), a Na^+^ K^+^- ATPase (*Atpα*), Contactin (*Cont*), Neuroglian (*Nrg*), Gliotactin (*Gli*), Melanotransferrin (*Mtf*), Varicose (*vari*), and Sinuous (*sinu*). Septate junctional complexes also contain cytoplasmic components important for their formation and/or function, such as Coracle (*cora*), which binds to the cytoplasmic domain of Nrx-IV (Izumi & Furuse, 2014) Vertebrate TJs and SJs have a number of components with homology to insect SJ proteins, suggesting evolutionary and functional conservation. Homologues of the insect claudins *sinu*, *megatrachea*, and *kune-kune* are present in vertebrate tight junctions, and vertebrate homologues of *Cont*, *Nrx-IV* and *Nrg* are present in SJs within glial sheets (Tepass et al., 2001).

The coordinated formation of epithelial barriers around stromal cells is a shared phenomenon critical to the function of diverse tissue types. Disruption of such epithelial barriers can lead to defects in tissue morphogenesis, organ function and cellular differentiation (Buckley & Turner, 2018). Loss of function mutations in the tight junction component *occludin* led to early neuronal differentiation and reduced proliferation of neural progenitors in mice (Bendriem et al., 2019). Similarly, knockdown of essential tight junction component claudin-6 in Xenopus led to morphogenetic defects in pronephros tubulogenesis and failure of duct and tubule cell differentiation in the developing kidney (Sun et al., 2015). In *Drosophila* lymph gland cells, disruption of the SJ led to increased crystal cell differentiation (Khadilkar & Tanentzapf, 2019) and in the *Drosophila* testis, disruption of SJs in the somatic cyst cells resulted in failure of germ line cells to differentiate (Fairchild et al., 2016).

**Figure 1.**
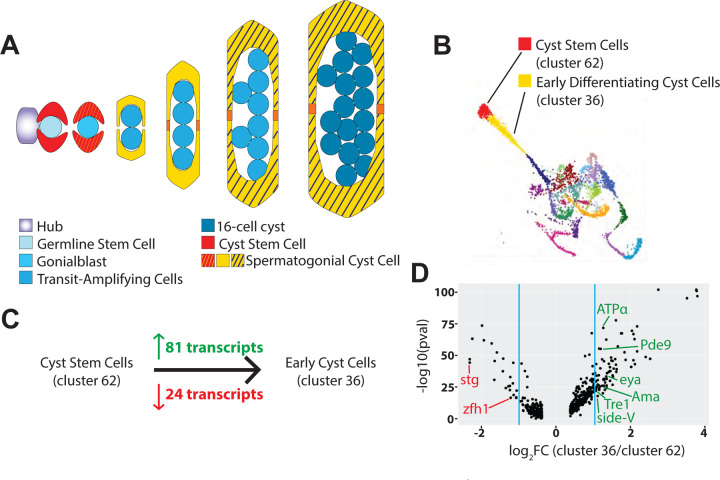
Differential expression analysis of testis snRNAseq data reveals genes upregulated as cyst stem cells differentiate. (A) Diagram of early stages in *Drosophila* spermatogenesis depicting cyst cell and germline lineages as a germline stem cell differentiates into late transit amplifying cells. (B) UMAP plot of the cyst cell lineage generated from testis snRNAseq data (Li et al., 2022), colored by clusters generated by Leiden 6.0 clustering. (C) Number of differentially expressed genes identified either as upregulated (green) or downregulated (red) between cluster 62 and cluster 36. (D) Volcano plot of genes differentially expressed between cluster 62 and cluster 36 with log_2_FC values greater than 1, generated with ASAP.

Drosophila spermatogenesis offers an excellent system for investigating the co-differentiation of epithelia and the cells they enclose. Male germ cells differentiate in a small mini-organ, the germ line cyst, in which a clone of germ cells descended from a single founder is enveloped by somatically derived cyst cells, which form a two cell squamous epithelium (Figure 1A) sealed by septate junctions (Fairchild et al., 2016). Male germ line stem cells, attached to hub cells at the testis apical tip, divide with oriented spindles so that one daughter remains attached to the hub and maintains germ line stem cell state, while the daughter displaced away and becomes a gonialblast (Fuller & Spradling, 2007). The male germ line stem cells are flanked by somatic cyst stem cells, which also divide to both self renew and produce non mitotic daughter cyst cells displaced away from the hub. Two somatic cyst cells flank and send out processes to enclose each gonialblast, pinching it off from its parent male germ line stem cell (Lenhart & DiNardo, 2015). These two cyst cells never divide again, but enclose and codifferentiate with the mitotic and meiotic progeny of the gonialblast throughout the entire process of spermatogenesis until sperm individualization. Genetic ablation of the cyst cell lineage leads to failure of the germline to differentiate beyond goniablast, indicating a critical role for this epithelial lineage in germline differentiation (Lim & Fuller, 2012).

During organogenesis and in tissue replenishment from adult stem cells, progenitor cells within many tissues undergo homeostatic programmed cell death (Fuchs & Steller, 2011; Ghose & Shaham, 2020). Rat oligodendrocyte precursors undergo massive cell death, with 50% of oligodendrocytes dying within 2–3 days of their birth (Barres & Raff, 1999) because they lack pro-survival signals from the axons they encapsulate. In the testis, programmed cell death has been observed in spermatogonial progenitors and meiotic spermatocytes of humans, mice and rats (Jeyaraj et al., 2003; Sinha Hikim & Swerdloff, 1999). In *Drosophila*, 20–30% of spermatogonial cysts are eliminated during homeostasis (Yacobi-Sharon et al., 2013), increasing to approximately 50% of cysts following protein starvation (Yang & Yamashita, 2015). Recent work has uncovered roles of somatic support cells in the cell death of germ line progenitor cells, as cyst cells in the *Drosophila* testis kill and phagocytose transit amplifying germline cells during homeostasis (Zohar-Fux et al., 2022) and protein starvation (Chiang et al., 2017). However, the initiating factors that induce this early germ cell death in the Drosophila testis have not been identified.

Here we show that septate junctions are required in cyst cells to ensure the survival of transit amplifying germ line cells and that this requirement depends on expression of the differentiation factor *bag of marbles (bam)*. Mining snRNAseq data from *Drosophila* testis (Li et al., 2022; Raz et al., 2023), we identified genes upregulated as cyst stem cells differentiate into early cyst cells and tested their function by cell type specific RNAi. For several of these upregulated genes, lowering function in cyst cells led to either overproliferation or loss of spermatogonia. Strikingly, loss of function in somatic cyst cells of any of several components of the *Drosophila* septate junction resulted in death of the germ cells they enclose at the transition from 4 cell to 8 cell cysts, corresponding the time at which septate junctions in cyst cells normally set up a barrier sealing off the germ cells from the testis milieu (Fairchild et al., 2016). The germ cell death occurs as the differentiation factor *bam* begins to accumulate in germ cells. Loss of function of *bam* protected germ cells from death when expression of septate junctions components was knocked down in the cyst cell lineage, suggesting that changes in germ cell state induced by *bam* make germline cells vulnerable to cell death following loss of barrier function in cyst cells.

## Results

### Differential expression analysis of testis snRNAseq data reveals transcripts upregulated as cyst stem cells initiate differentiation

Differential expression analysis using ASAP (Gardeux et al., 2017) of clusters of cells from *Drosophila* testis snRNA seq data (Li et al., 2022) identified transcripts upregulated as cyst stem cells differentiate into early cyst cells. Cluster 62 was previously identified as containing cyst stem cells and cluster 36 as containing the earliest differentiating cyst cells (Raz et al., 2023). ASAP identified 24 transcripts downregulated by at least 2-fold in cluster 36 compared to cluster 62. These included the mitotic marker *string* (*stg*) and the Jak/STAT-target *zfh1* (Leatherman & Dinardo, 2008) required for cyst stem cell maintenance (Figure 1C, D), consistent with the assignment of cluster 62 as cyst stem cells and cluster 36 as early differentiating cyst cells. Reciprocally, ASAP identified 81 transcripts upregulated more than 2-fold in cluster 36 compared to cluster 62, indicating transcription of these genes increases as cyst stem cells differentiate into early cyst cells (Figure 1C, D, Table S1). The 81 upregulated transcripts included *eyes absent* (*eya*), consistent with the increase in Eya protein observed in nuclei of cyst cells associated with transit amplifying spermatogonia and later stage cysts observed by immunofluorescence (Fabrizio et al., 2003).

### Cell type-specific RNAi of upregulated transcripts identifies genes required in cyst cells to limit germ cell proliferation

**Figure 2.**
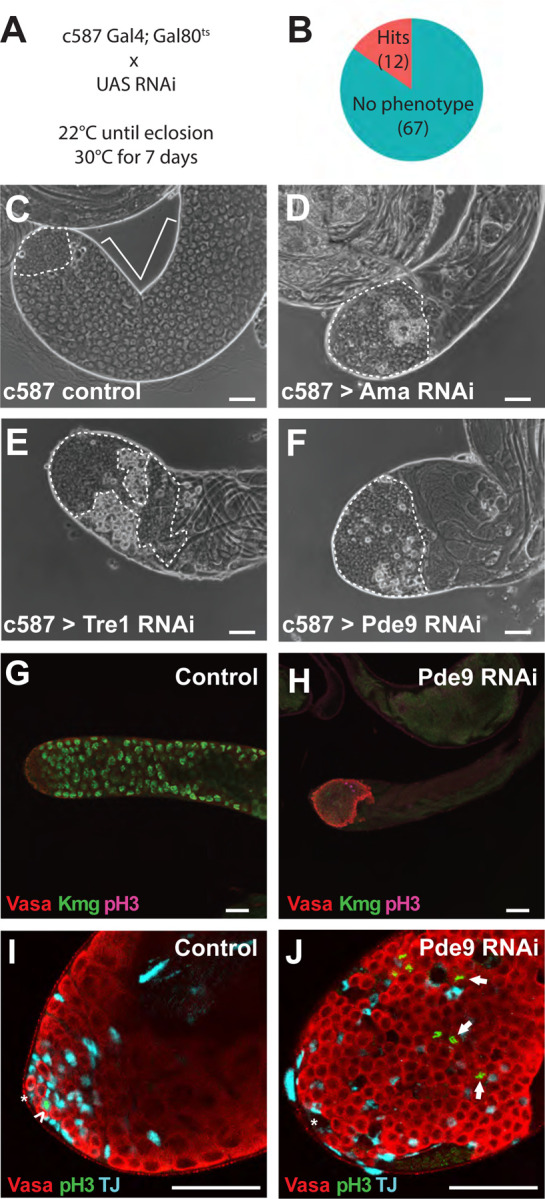
Cyst cell-specific RNAi screen identified genes required in cyst cells for early germ cell differentiation. (A) Fly crosses and temperature shift regimen employed to induce cyst cell-specific RNA knockdown of candidate genes. (B) Pie chart indicating number of genes for which knockdown in cyst cells by RNAi produced a phenotype as assessed by phase-contrast microscopy of dissected testis tissue. (C-F) Phase-contrast images of the apical region of testes from male *c587Gal4, Gal80*^*ts*^ flies shifted to 30°C for 7 days with (C) no RNAi transgene or an RNAi transgene targeting (D) *Ama*, (E) *Tre1*, or (F) *Pde9*. (G, H) Immunofluorescence images of the apical region of testes stained with (red) anti-Vasa and (green) anti-Kmg and (pink) anti-ph3 antibodies from male *c587Gal4, Gal80*^*ts*^ flies shifted to 30°C for 7 days that had (G) no RNAi transgene or (H) an RNAi transgene targeting *Pde9*. (I, J) Immunofluorescence images stained with (red) anti-Vasa, (green) anti-PH3 and (teal) anti-TJ from *c587Gal4, Gal80*^*ts*^ male flies shifted to 30°C for 7 days that had (I) no RNAi transgene or a (J) *Pde9* RNAi transgene. Dotted outline: spermatogonia, bracket: spermatocytes. Scale bars: 50μm.

A cyst cell RNAi screen to knock down expression of transcripts upregulated as cyst stem cells differentiate into early cyst cells revealed both known and novel genes required in cyst cells for early steps in germ cell differentiation. To knock down gene function in cyst cells, flies carrying transgenes encoding *c587Gal4* and a temperature-sensitive Gal4 repressor, *tub-Gal80*^*ts*^, were crossed to fly lines carrying UAS-RNAi constructs targeting 79 of the 81 upregulated genes (Table S1). *c587Gal4* drives expression in the cyst cell lineage (Kai & Spradling, 2003), as well as in other somatic tissues throughout the fruit fly. The progeny were raised to adulthood at 22^o^C to allow normal testis development, then shifted to 30^o^C to initiate knock down (Figure 2A). Following 7 days of knock down at 30^o^C, testes were dissected and examined by phase contrast light microscopy to identify effects on germ cell differentiation. Testes from control flies carrying the *c587Gal4* expression driver and *tub-Gal80*^*ts*^ but lacking the RNAi line and raised under the same temperature shift regimen resembled wild type, with small cells (dotted outline) at the apical tip including germline stem cells and transit-amplifying spermatogonia, many larger cells in spermatocyte stages (bracketed) further from the tip (Figure 2C), and elongated spermatid bundles resulting from germ cell differentiation extending apically from the terminal end of the testes. Targeting 79 candidate genes with a total of 255 RNAi lines (Table 1) under these conditions identified 12 genes where knock down in the cyst cell lineage showed clear phenotypes when assessed by phase light microscopy (Figure 2B).

Knock down of *Ama*, *Tre1* or *Pde9* in cyst cells resulted in increased numbers of small cells at the tip of the testis compared to control, with no or few spermatocytes remaining after 7 days at 30^o^C (Figures 2D–F). Immunofluorescence staining with anti-Vasa to mark germ cells and anti-TJ to mark the nuclei of cyst stem cells and early cyst cells confirmed that testes in which Pde9 was knocked down in the cyst cell lineage by RNAi had a large cluster of small germ cells at the apical tip and lacked cells expressing the spermatocyte marker Kmg (Figure 2G, H). In control testes, staining for the mitotic marker phospho-histone H3 (pH3) only showed pH3 positive germ cells (marked by anti-Vasa) within a few cell diameters of the hub (Figure 2I). In contrast, testes lacking Pde9 function in cyst cells showed pH3 positive germ cells in mitosis much farther from the hub. These were commonly singlets, doublets, or only very small clusters, indicating that the germ cells were proliferating as gonialblasts or early spermatogonia (Figure 2J), rather than as large cysts of germ cells undergoing synchronized divisions as in *bam* mutant males.

### Function of Septate Junction (SJ) components is required in somatic cyst cells for survival of late transit amplifying spermatogonia.

**Figure 3.**
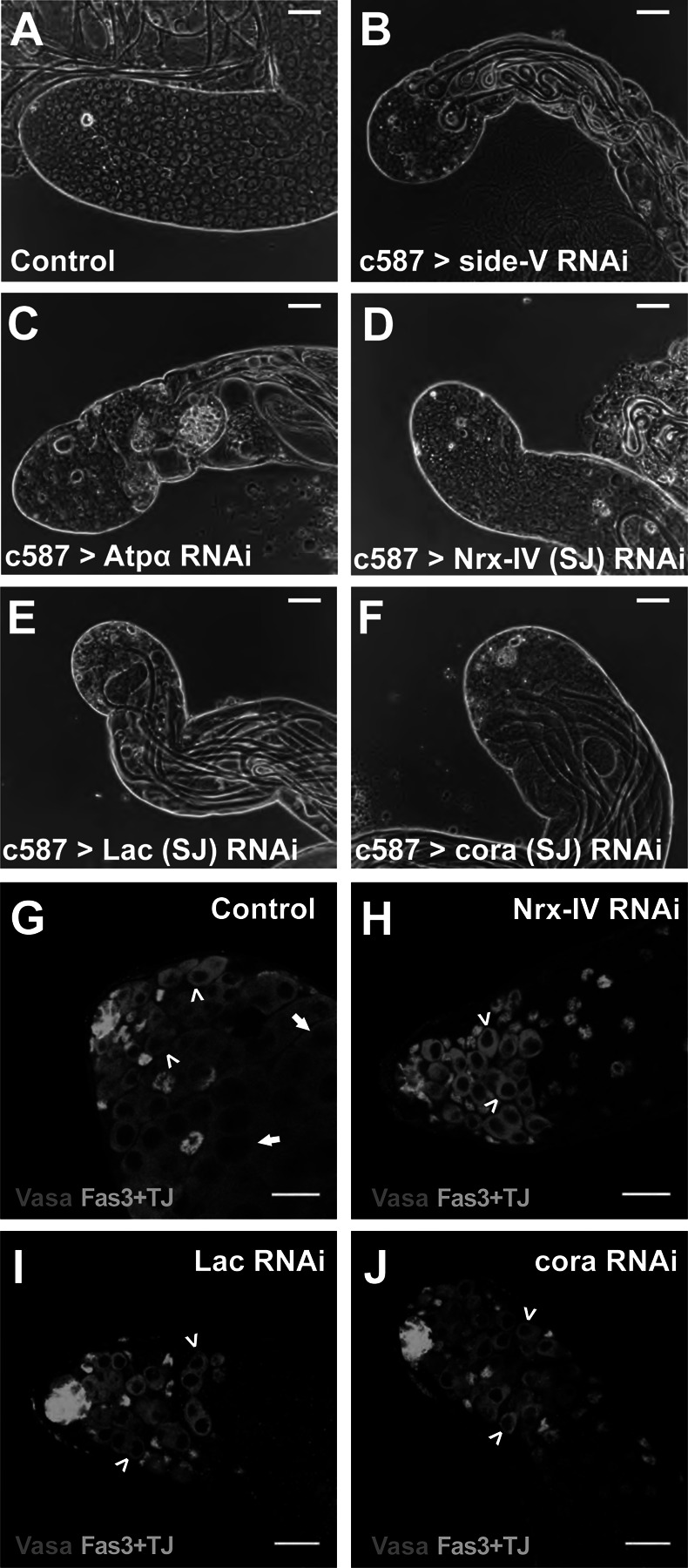
Knockdown of Septate Junction components in cyst cells resulted in loss of mid stage transit amplifying germ cells. (A-F) Phase-contrast images of the apical region of testes from male *c587Gal4, Gal80*^*ts*^ flies shifted to 30°C for 7 days that had (A) no RNAi transgene or an RNAi transgene targeting (B) *side-V*, (C) *Atpα*, (D) *Nrx-IV*, (E) *Lac* or (F) *cora*. (G-J) Immunofluorescence images of the apical region of testes stained with (teal) anti-Fas3 and anti-TJ and (red) anti-Vasa antibodies from male *c587Gal4*, *Gal80*^*ts*^ flies shifted to 30°C for 7 days that had (G) no RNAi transgene, or an RNAi transgene targeting (H) *Nrx-IV*, (I) *Lac*, or (J) *cora*. Arrowheads: spermatogonia; Arrows: spermatocytes. Scale bars: 50μm.

In contrast, knock down of the motor axon guidance receptor *side-V* (Sink et al., 2001) or the Septate Junction (SJ) component *Atpα* in the cyst cell lineage resulted in testes with only a few small cells remaining at the testis tip (Figure 3B, C), compared to control testes, which contained many germ cells at different stages of differentiation (Figure 3A). Under the knock down conditions assessed, loss of function of *side-V* or *Atpα* in the cyst cell lineage resulted in testis with elongated spermatid bundles (from before the temperature sift), but lacking spermatocytes and early spermatid stages. Notably, a limited number of small cells resembling germ cells remained clustered at the apical tip.

Further investigation by RNAi showed that knock down in cyst cells of several other septate junction components, including *Neurexin-IV* (*Nrx-IV*), *Lachesin* (*Lac*), and *coracle* (*cor*), resulted in phenotypes similar to knock down of *Atpα*, assessed by phase contrast microscopy (Figure 3D–F), suggesting that canonical septate junctions in cyst cells play an important role in supporting survival of germ cells in the spermatogonial transit amplifying divisions. Immunofluorescence staining with anti-Vasa to mark germ cells and anti-TJ to mark the nuclei of cyst stem cells and early cyst cells showed that control testes from flies carrying *c587Gal4* and *tub-Gal80*^*ts*^ but lacking the RNAi construct subjected to the knock down temperature regime had abundant early germ cells and spermatocytes (Figure 3G). In contrast, by 7 days after the shift to 30^o^C to initiate knock down, loss of function of the core transmembrane SJ components Neurexin-IV (*Nrx-IV*) or *Lachesin* (*Lac*) or of the cytoplasmic SJ component *coracle* (*cor*) in the cyst cell lineage resulted in the loss of early germ cells undergoing spermatogonial divisions. In all three cases, cyst cell knockdown of the septate junction components resulted in testes that retained a small group of germ cells at the tip of the testis (Figure 3H–J), but lacked spermatocytes. Strikingly, most testes did not have overproliferating germline cysts.

**Figure 4.**
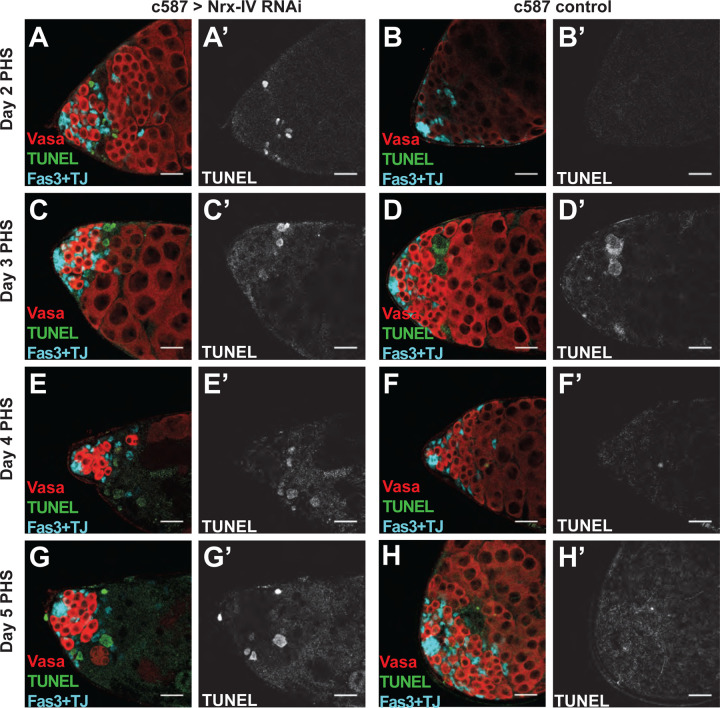
Loss of Nrx-IV in cyst cells results in acute germ cell death at mid transit amplifying stages. (A-H) Immunofluorescence images of the apical region of testes stained with (teal) anti-Fas3 and anti-TJ, and (red) anti-Vasa antibodies along with (green/white) TUNEL from male *c587Gal4, Gal80*^*ts*^ flies shifted to 30°C for the specified number of days. Control flies with no RNAi construct were shifted to 30°C for (B) 2, (D) 3, (F) 4 or (H) 5 days prior to dissection. Nrx-IV knockdown flies, which had the RNAi transgene targeting Nrx-IV, were shifted to 30°C for (A) 2, (C) 3, (E) 4 or (G) 5 days prior to dissection. Scale bars: 50μm.

Analysis of time points soon after induction of RNAi revealed that spermatogonia were lost to cell death when function of the SJ component Nrx-IV was knocked down in somatic cyst cells. An increase in the number of TUNEL-positive cells compared to controls appeared as early as 2 days post induction of RNAi against Nrx-IV in cyst cells (Figure 4A, B). The TUNEL-positive cells were located in a zone of germ cell death in the region where late transit-amplifying spermatogonial germ cells normally reside.

Testes observed at later time points after Nrx-IV knockdown also had increased TUNEL staining at the testis tip compared to control testes raised with the same temperature regimen. At day 3 post onset of Nrx-IV knockdown, large, maturing spermatocytes were adjacent to spermatogonia (Figure 4C), suggesting that germ cells beyond the zone of cell death, presumably generated prior to the knockdown, continued to differentiate. Prolonged knockdown resulted in morphological changes, including a thinner testis shape (Fig 4C–H), likely due to decline in the number of differentiating spermatocytes. By day 5 of Nrx-IV knockdown (Figure 4G) testes resembled day 7 of Nrx-IV knockdown (Figure 3H), with a small group of germ cells remaining at the testis tip.

### Nrx-IV is required in the cyst cell lineage for germ cells to progress beyond the 4-cell transit amplifying stage.

**Figure 5.**
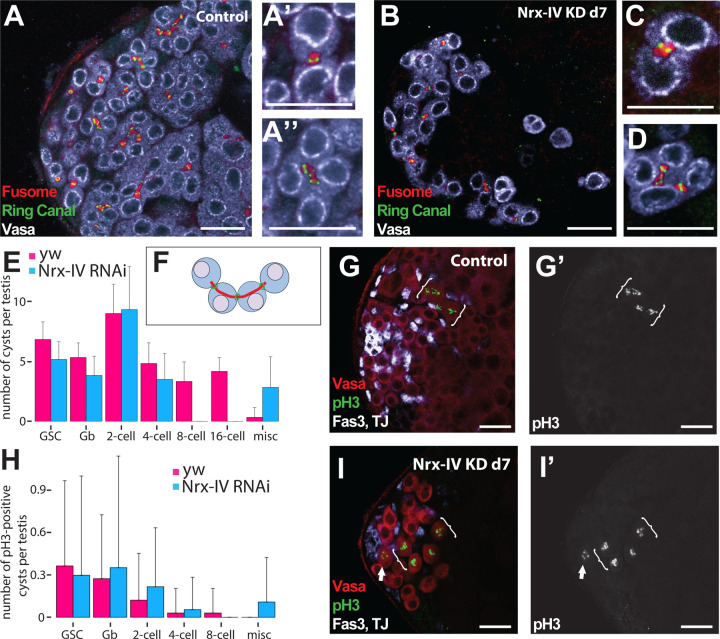
Nrx-IV is required in cyst cells for germ cells to progress beyond the 4-cell stage. (A-D) Immunofluorescence images of the apical region of testes stained with (red) anti-Hts, (green) the ring canal marker anti-cindr, and (white) anti-Vasa antibodies from male *c587Gal4, Gal80*^*ts*^ flies shifted to 30°C for 7 days that had (A) no RNAi transgene or (B-D) an RNAi transgene targeting *Nrx-IV*. Higher magnification pictures show (A’ and C) 2-cell and (A’’ and D) 4-cell spermatogonial cysts. (E) Quantification of the number of germline stem cell (GSC), gonialblast, 2-cell, 4-cell, 8-cell, 16-cell or miscellaneous cysts per testis (n=7 for yw; n=6 for *Nrx-IV* RNAi) and (H) quantification of the number of pH3-positive cysts per testis (n=33 for yw; n=36 for *Nrx-IV* RNAi) from *c587Gal4, Gal80*^*ts*^ males shifted to 30°C for 7 days with (blue) no RNAi transgene or (red) an RNAi transgene targeting *Nrx-IV*. (F) Diagram of a 4-cell transit amplifying cyst with fusome in green and ring canals in red. (G, I) Immuno-fluorescence images of the apical region of testes stained with (white) anti-Fas3 and anti-TJ, (red) anti-Vasa and (green) anti-pH3 antibodies from *c587Gal4, Gal80*^*ts*^ male flies shifted to 30°C for 7 days that had (G) no RNAi transgene or (I) an RNAi transgene targeting *Nrx-IV*. Arrow: dividing germline stem cell; Brackets: cysts of mitotic 4-cell transit amplifying cells. Scale bars: 50μm.

Staining of ring canals and fusome to allow precise quantification and analysis of the stage of transit amplifying germ line cysts revealed that early spermatogonial cysts were present in normal numbers after knockdown of Nrx-IV in somatic cyst cells, but that the majority of spermatogonia did not survive beyond the 4-cell stage. During the transit amplifying spermatogonial divisions, germ cells undergo incomplete cytokinesis, resulting in cysts of interconnected germ cells that are mitotic sisters, connected by ring canals. The fusome, a cytoplasmic, membranous structure, runs through these ring canals within a germline cyst, grouping the germ cells together in a cluster (Hime et al., 1996). The germ cells in 2-cell stage cysts have one ring canal strung on a short fusome connecting the two germ cells (Figure 5A’). 4-cell cysts have 3 ring canals strung along a curved fusome (Figure 5A”, 5D). Cysts at the 8- and 16-cell stages have 7 and 15 ring canals respectively, linked by a branched fusome. Immunofluorescence staining of ring-canal and fusome components in testes in which function of Nrx-IV had been knocked down in the somatic cyst cell lineage for 7 days revealed a lack of cysts with 8 or 16 germ cells. 4-cell cysts were as common in the knock down as in control testes subjected to the same temperature shift regimen (Fig 5B–E). However, many 4-cell cysts in Nrx-IV knock down testes had aberrant fusomes that did not correctly span all three ring canals (Fig 5D). In the 6 Nrx-IV knock down testes analyzed by ring canal-fusome staining, no 8-cell cysts were identified (Figure 5E), whereas twenty 8-cell cysts were identified in the 6 control testes stained in parallel. Additionally, testes in which NrxIV had been knocked down in the cyst cell lineage for 7 days contained germ cells that did not resemble germ cells observed in control testes, including a small percentage of escaper cysts that contained more than 16 cells (i.e. a 22-cell cyst) and isolated pairs of germ cells that had more than one ring canal, suggesting the two cells may have broken off from a 4-cell cyst.

Staining for the mitotic chromosome marker pH3 to score cysts of germ cells undergoing synchronous mitosis indicated that spermatogonia up to the 4-cell stage were able to enter the mitotic program prior to undergoing cell death under conditions where Neurexin-IV had been knocked down in somatic cyst cells. Quantification of germ cells undergoing mitotic division based on immunofluorescence staining with anti pH3 showed GSCs, Gbs, and germ cells in 2-cell or 4-cell cysts undergoing mitosis in testes where Nrx-IV had been knocked down in the cyst cell lineage, as in controls (Figure 5G–I). However, consistent with the lack of 8-cell cysts indicated by the analysis of ring canals and fusome above, no cysts with 8 germ cells in mitosis were detected in the 37 Nrx-IV knock down testes examined (Figure 5H).

### Cyst cells persist in the absence of Nrx-IV, but activate the Jnk pathway and germ cells undergo omi-dependent death.

**Figure 6.**
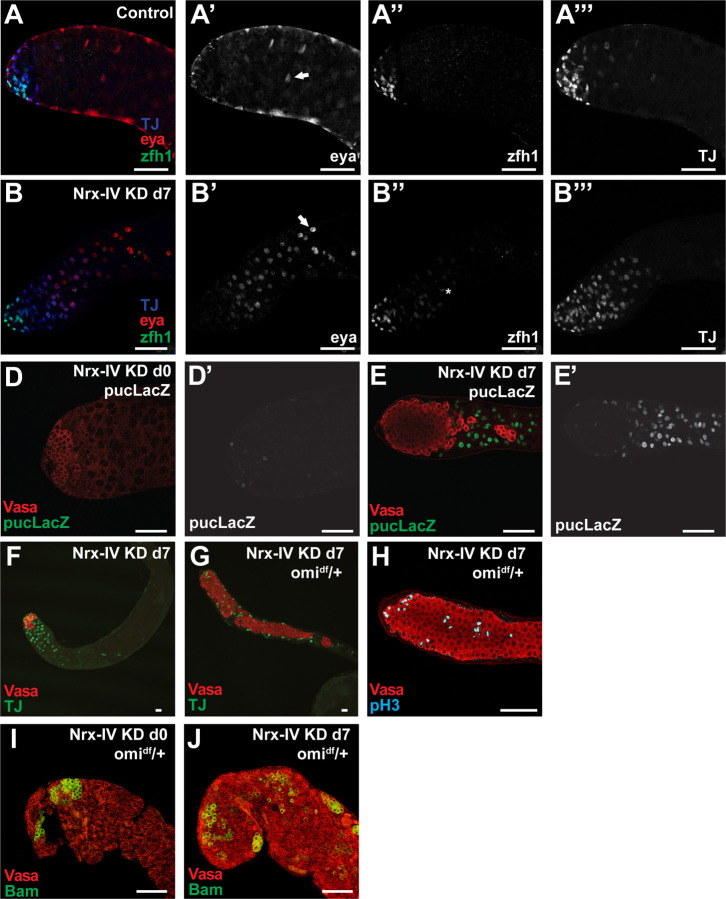
Knockdown of *Nrx-IV* results in activating the JNK pathway and *omi*-dependent germ cell death. (A, B) Immunofluorescence images of the apical region of testes stained with (red) anti-Eya, (green) anti-Zfh1 and (blue) anti-TJ antibodies from male *c587Gal4, Gal80*^*ts*^ flies shifted to 30°C for 7 days that had (A) no RNAi transgene or (B) an RNAi transgene targeting *Nrx-IV*. (D, E) Immunofluorescence images of the apical region of testes stained with (red) anti-Vasa and (green/white) anti-LacZ antibodies from male *c587Gal4, Gal80*^*ts*^ flies that had a *pucLacZ* transgene and an RNAi transgene targeting *Nrx-IV* shifted to 30°C for (D) 0 days as control or (E) 7 days to induce knockdown. (F, G) Immunofluorescence images of the apical region of testes stained with (red) anti-Vasa and (green) anti-TJ antibodies from male *c587Gal4, Gal80*^*ts*^ flies shifted to 30°C for 7 days that had an RNAi transgene targeting *Nrx-IV* with (F) wild-type *omi* alleles or (G) heterozygous *omi*^*df*^/+. (H) Immunofluorescence images of the apical region of testes stained with (red) anti-Vasa and (teal) anti-pH3 antibodies from male *c587Gal4, Gal80*^*ts*^ flies shifted to 30°C for 7 days that had an RNAi transgene targeting Nrx-IV with heterozygous omi^df^/+. (I, J) Immunofluorescence images of the apical region of testes stained with (red) anti-Vasa and (green) anti-Bam antibodies from male *c587Gal4, Gal80*^*ts*^ flies that had an RNAi transgene targeting *Nrx-IV* shifted to 30°C for (I) 0 days as control or (J) 7 days to induce NrxIV knockdown. Arrows indicate eya positive cyst cells. Scale bars: 50μm.

Loss of Nrx-IV did not grossly affect the ability of cyst stem cells to self-renew or differentiate into early cyst cells. While Nrx-IV knockdown in the cyst cell lineage under control of *c587-Gal4* led to loss of germ cells, immunofluorescence staining confirmed that cyst stem cells and cyst cells lacking Nrx-IV near the apical tip of the testis expressed the nuclear marker *traffic jam (tj).* In addition, *eyes absent (eya*) protein was detected in nuclei of cyst cells away from the testis tip, as in controls (Figure 6AB). Cyst stem cells near the apical tip of testes in which function of Nrx-IV had been knocked down by RNAi under control of *c587Gal4* robustly expressed Zfh1, a readout of the response to Jak-Stat signaling characteristic of cyst stem cells in wildtype (Fig 6A, B). Normally the level of Zfh1 protein detected by immunofluorescence staining in cyst cell nuclei falls in cyst cells accompanying 2, 4, and 8 cell cysts, with little or no signal detected once the cysts reach spermatocyte stages (Leatherman & Dinardo, 2008) and Figure 6A) However, in testes where function of Nrx-IV had been knocked down in cyst cells, low levels of Zfh1 staining persisted in differentiating cyst cells , even considerably away from the hub (Figure 6B, asterisk). Although based on the markers Eya and Zfh1, somatic cyst cells persisted after the death of late spermatogonia in Nrx-IV knockdown testes, the cyst cells remaining after germ cell death expressed high levels of *pucLacZ*, a readout of JNK pathway activation not normally expressed at high levels in cyst cells (Figure 6D,E).

Death of spermatogonia in 8 cell cysts caused by loss of function of Nrx-IV in cyst cells likely occurred by the alternative germ cell death pathway that normally prunes spermatogonial cysts during homeostasis. Knock down of Nrx-IV in cyst cells combined with lowered levels *omi*, a component of an alternative cell death pathway known to contribute to death of *Drosophila* spermatogonia downstream of cyst cell action in response to nutritional limitation (Yacobi-Sharon et al., 2013), resulted in a large increase in the number of surviving germ cells. Strikingly, the germ cells rescued from death in after knockdown of Nrx-IV in cyst cells in the *omi/+* background filled the testis and remained small, indicating lack of differentiation (Figure 6G). The cyst cell nuclei associated with these rescued spermatogonia remained positive for the early cyst cell marker Tj (Figure 6G), indicating that they too did not progress to differentiation, as Tj normally turns off in cyst cells soon after the germ cells they enclose become spermatocytes (Figures 3G, 6A’’). Staining for the mitotic marker pH3 indicagted that the rescued germ cells divided in small clusters or singlets (Figure 6H), consistent with a mixed identity of gonialblasts and transit amplifying cells. Consistent with this, immunofluorescent staining for Bam revealed that most of the overproliferating germ cells were Bam negative (Figure 6I, J) with a small number of Bam positive germline clusters scattered down the testis. In contrast, in *omi*/+ testes in which Nrx-V had not been knocked down, Bam positive cells were restricted to a ring near the apical tip of the testis (Figure 6I), as in wildtype.

### Death of transit amplifying spermatogonia due to loss of Nrx-IV function in cyst cells requires the germ line differentiation factor Bam

**Figure 7.**
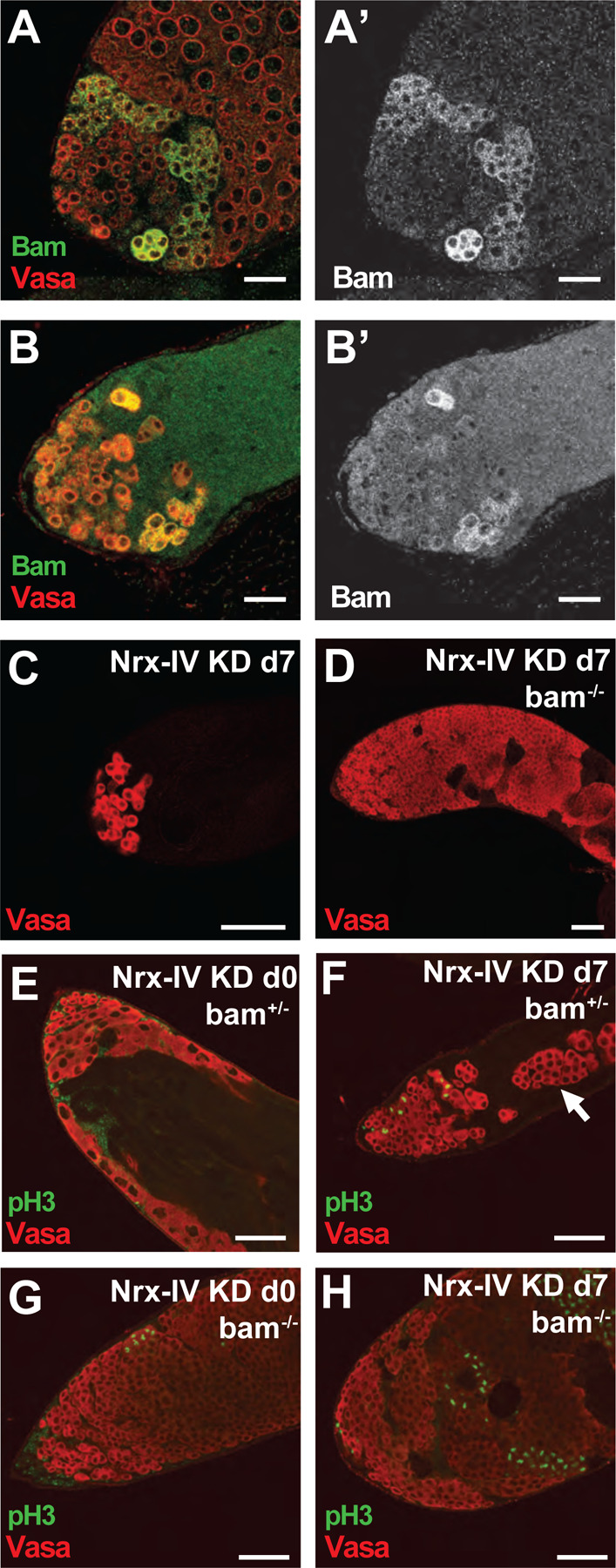
Bam is required for Nrx-IV-deficient cyst cell-mediated TA cell death. (A, B) Immunofluorescence images of the apical region of testes stained with (red) anti-Vasa and (green) anti-Bam antibodies from male *c587Gal4, Gal80*^*ts*^ flies shifted to 30°C for 7 days that had (A) no RNAi transgene or (B) an RNAi transgene targeting *Nrx-IV*. (C, D) Immunofluorescence images of the apical region of testes stained (red) anti-Vasa from male *c587Gal4, Gal80*^*ts*^ flies that had an RNAi transgene targeting *Nrx-IV* and were shifted to 30°C for 7 days that were (C) wildtype for *bam* or (D) homozygous *bam*^−/−^. (E-H) Immunofluorescence images of the apical region of testes stained (red) anti-Vasa and (green) anti-pH3 antibodies from male *c587Gal4, Gal80*^*ts*^ flies carrying an RNAi transgene targeting *Nrx-IV* shifted to 30°C for (E, G) 0 as control or (F, H) 7 days that were (E, F) heterozygous *bam*^+/−^ or (G, H) homozygous *bam*^−/−^. Arrow: large cyst of spermatogonia. Scale bars: 50μm.

The time at which spermatogonia began to show abnormalities (4-cell cysts) then died (by the 8-cell cyst stage) in Nrx-IV knock down testis corresponded to when the germ cell differentiation factor Bag-of-Marbles (Bam) becomes expressed. In wildtype testes, Bam protein begins to be detected by immunofluorescence staining in 4 cell cysts, with levels detected increasing in 8-cell stage spermatogonial cysts (Figure 7A). Insco *et al.* (2009) showed that immunodetection of Bam protein remained high in early 16 cell cysts, then the protein abruptly disappeared after completion of premeiotic S phase (Insco et al., 2009). In testes in which Nrx-IV was knocked down in cyst cells, some germ line cysts at the edge of the death zone showed immunofluorescence signal for Bam protein, but the number of Bam positive cysts per testis was always low (Figure 7B).

The death of mid-stage transit amplifying spermatogonia following knock down of Nrx-IV in cyst cells appears to depend on the expression of Bam in the germline. Testes from males mutant for *bam* in which Nrx-IV was knocked down in cyst cells by RNAi had a large number of proliferating spermatogonia, in contrast to the small number of early germ cell cysts in males with Nrx-IV knockdown alone (Figure 7C, D). Even partial decrease in Bam levels improved germ cell survival. A moderate increase in the number of germ cells was observed in testes when Nrx-IV was knocked down by RNAi in bam^+/−^ heterozygotes, compared to Nrx-IV knockdown alone (compare Vasa positive cells in Figures 7C and 7F), with proliferating escaper cysts observed beyond the group of germ cells at the tip of the testis (Figure 7F). Immunofluorescence staining for pH3 in *bam*^*−/−*^ homozygotes following Nrx-IV knockdown revealed large testes filled with more germline cells proliferating in synchrony relative to in *bam*^−/−^ homozygotes prior to knock down (Figure 7G, H), unlike Nrx-IV knockdown in *omi*/+ flies, which resulted in asynchronous germ cell proliferation (Figure 6H).

## Discussion

Our findings reveal that functional septate junctions in cyst cells are required for the survival of germ cells that have initiated differentiation as a result of activation of the germline differentiation factor Bam. This stage specific loss of transit amplifying germ cells likely involves the non-canonical cell death pathway that normally acts to prune proliferating spermatogonia (Yacobi-Sharon et al., 2013), as lowering function of *omi* suppressed the germ cell death induced by loss of septate junctions in somatic cyst cells. Our results raise the possibility that as germ cells turn on the differentiation factor *bam*, a surveillance system that detects whether the cyst is sealed off from the tissue environment by functional occluding junctions becomes activated. In this model, if the two enveloping cyst cells have not established a seal by the time *bam* protein reaches the threshold sufficient to trigger progression to differentiation in the germ cells they enclose, the surveillance system triggers germ cell death.

Previous work on septate junctions in *Drosophila* spermatogonial cysts revealed that the two somatic cyst cells begin to set up an occluding barrier around the germ cells they enclose at the 4-cell transit amplifying stage (Fairchild et al., 2016). This timing is consistent with when we began to observe defects in germ cell cysts following knockdown of septate junction components in cyst cells. Specifically, knockdown of Nrx-IV spared 2-cell cysts but led to a complete depletion of all germ cell types beyond the 4-cell transit amplifying stage. This coincidence in timing, along with the similar phenotypes caused by knockdown of other septate junction components, is consistent with a requirement for intact occluding junctions in cyst cells (rather than other roles of Nrx-IV) for survival of differentiating germ cells.

The timing of germ cell death induced by loss of function of Nrx-IV in cyst cells correlated with the onset of accumulation of Bam protein in the germ cells. Strikingly, loss of function of *bam* rescued the Nrx-IV knockdown cell death phenotype, leading to overproliferation of undifferentiated germ cells. In addition, lowering the gene dosage of *bam* (in *c587Gal4, Gal80*^*ts*^*; bam*^*86*^*/+; UAS Nrx-IV RNAi* males) allowed germ cell cysts to survive to later stages of proliferation than similar males wild type for *bam*. Based on these results, we suggest that germ cells that have accumulated *Bam* protein to a critical level are targeted to die if the cyst cells lack functional septate junctions, while gonialblasts and early transit amplifying spermatogonia that have not yet accumulated Bam protein are spared.

Septate junctions were previously implicated in differentiation of transit amplifying cells (Fairchild et al., 2016). Our result that reducing components of the germ cell death pathway (*omi*^+/−^ flies) while knocking down expression of Nrx-IV in cyst cells rescued the germ cell death but did not result in germ cell differentiation confirmed this finding , suggesting that Nrx-IV in cyst cells likely has at least two roles, required both for survival of germ cells that have embarked on the differentiation program triggered by *bam* and, if the germ cells escape death, for differentiation of spermatogonia into spermatocytes. Future work to uncover the surveillance mechanisms that detect whether transit amplifying cells in which Bam protein has begun to accumulate are enclosed by cyst cells with functional occluding junctions, as well the downstream pathways dependent on function of septate junction components in cyst cells that are required for germ cell differentiation, will reveal how loss of septate junctions in cyst cells can result in two distinct outcomes, depending on the state of the germline.

The death of transit amplifying germ cells that have initiated differentiation caused by loss of Nrx-IV function in cyst cells appears to involve a cell death pathway used to prune germ cell cysts under homeostasis or starvation. In *Drosophila* testes, 20% of transit-amplifying germ cell cysts are eliminated during normal homeostasis, and germ cell loss is greatly increased under conditions of protein starvation (Yacobi-Sharon et al., 2013; Yang & Yamashita, 2015). This pruning of differentiating cysts did not involve the canonical caspase based cell death pathway (Florentin & Arama, 2012) but depended on high levels of a mitochondrial protease, Omi (Yacobi-Sharon et al., 2013). Strikingly, loss of one copy of *omi* was also sufficient to prevent most cell death in testes lacking Nrx-IV in cyst cells. It is tempting to speculate that the normal Omi-dependent pruning of germ cell cysts under homeostasis or starvation conditions may be due to the surveillance checkpoint that detects intact occluding junctions we propose above, so that only transit amplifying cysts with properly formed septate junctions survive. If germ cells cannot differentiate but continue to proliferate without proper enclosure by cyst cells the surveillance mechanism we propose may provide an important safeguard against aberrant overproliferating cells that could progress to germ cell tumors. As occluding junctions between somatic support cells that isolate certain germ cell stages from the body occur in many animal species (Luaces et al., 2023) this may be an evolutionarily conserved mechanism.

The Jun-kinase (JNK) pathway is activated, marked by upregulation of the reporter *puc-lacZ,* in cyst cells following protein starvation (Yang & Yamashita, 2015), a condition in which cyst cells kill germ cells. Cyst cells lacking function of apical polarity Par proteins also activate the JNK pathway, leading to death of the early spermatocytes the cyst cells enclose (Brantley & Fuller, 2019). Although *puc-lacZ* was also upregulated in cyst cells lacking Nrx-IV, we cannot distinguish whether activation of the JNK pathway in cyst cells lacking Nrx-IV facilitates the killing of the associated transit amplifying germ cells or whether the activation of the JNK pathway in cyst cells is a response to the death or loss of the germ cells they normally enclose.

While the genes required for formation of septate junctions have been investigated extensively in *Drosophila* early embryonic development (Baumgartner et al., 1996; Ward et al., 1998), much remains unknown about the initial molecular events that promote septate junction formation. In *Drosophila* embryos, SJ proteins are expressed before embryonic stage 12, yet they do not form stable complexes until mid-to-late stage 13 (Oshima & Fehon, 2011). The initiating factor for SJ complex formation has yet to be identified. In *Drosophila* testes, Nrx-IV protein is expressed in both cyst stem cells and differentiating cyst cells, while functional septate junctions able to occlude passage of 10 kDa Dextran into cysts were not established until cysts had reached the 4–8 germ cell stage (Fairchild et al., 2016). Following from our snRNAseq analysis, it is possible that ATPα is a limiting factor, with transcriptional upregulation in early cyst cells triggering initiation of septate junction formation.

Our results underscore the utility of using stage specific gene expression changes revealed in analysis of existing scRNA or snRNA seq data from tissues containing cells in a differentiation sequence to guide small-scale, targeted functional screens. This approach could be particularly useful for identifying functional genes in rare cell types within a tissue. It may also be useful for probing how gene expression changes in one cell type can affect differentiation of partner cells of different origin within tissues, for example changes in epithelial cells affecting the state of underlying mesenchyme in an organ. One of the genes our screen identified as required in cyst cells for germ line differentiation, side-V, is a member of the Side family, a group of proteins that contain Immunoglobulin (Ig) domains and act as receptors for Beaten Path ligands (Zinn & Ozkan, 2017). Both the Beaten Path ligands and the Side receptors are critical for accurate projection of motor neuron axons into muscles (Li et al., 2017; Sink et al., 2001). As members of the Beaten Path family are expressed in both cyst cells and early germ cells in *Drosophila*, they are good candidates for further investigation. While we did not detect phenotypes in RNAi crosses for many of the genes that were upregulated as cyst cells differentiate, this could be in part due to failure of the RNAi lines tested to knock down mRNAs strongly enough to reveal gene function (Dietzl et al., 2007; Perkins et al., 2015). Further work using stronger Gal4 drivers and expression systems, loss of function mutations, or tissue-specific CRISPR to induce cell type specific loss of gene function (Port & Boutros, 2022) could lead to the identification of functional roles for additional genes upregulated as cyst stem cells differentiate.

## Methods

### Fly strains and husbandry

*Drosophila* strains were raised in standard molasses medium at 25°C unless otherwise noted. The following *Drosophila* strains were used: *w*^*1118*^ as wild-type; *c587-Gal80*^*ts*^; RNAi fly stocks were obtained from the Vienna *Drosophila* Resource Center (VDRC) (Dietzl et al., 2007) and further detailed in Table 5.1.

### RNAi knockdown protocol

RNA interference (RNAi) knock-down (KD) experiments were carried out by crossing males with a *UAS-RNAi hairpin* transgene (VDRC) to virgin females of *c587Gal4;ScO/CyO;Gal80*^*ts*^. Crosses were set up at 22°C and young male progeny were shifted to 30°C to allow inhibition of *Gal80* and expression of the RNAi hairpin for the indicated number of days.

### Immunofluorescence staining

For wholemount staining, testes from males were dissected in 1X phosphate-buffered saline (PBS) and incubated with 4% formaldehyde for 20 minutes at room temperature. After fixation, the testes were washed once in PBST (PBS with 0.1% Triton X-100) and permeabilized by incubation with PBS with 0.3% Triton X-100 and 0.6% sodium deoxycholate for 30 minutes at room temperature. After permeabilization, testes were washed once in PBST and blocked for 30 minutes with PBST with 3% bovine serum albumin (BSA), then incubated overnight at 4C with desired primary antibodies in PBST with 3% BSA. After overnight incubation in primary antibody, testes were washed three times with PBST, incubated with secondary antibodies conjugated with Alexa fluorophores (Alexa Fluor-488, −568, −647 from Molecular Probes were used) for 2 hours at room temperature in the dark while rocking, then washed three times in PBST and mounted on glass slides with mounting medium with DAPI (VECTASHIELD, Vector Labs, Cat# H-1200).

The sources and dilutions of primary antibodies used were as follows: anti-Vasa (goat, 1:100; Santa Cruz Biotechnology, Cat# dc-13); anti-TJ (guinea pig, 1:10,000, a gift from D. Godt, University of Toronto, Canada); anti-Eya (mouse, 1:200, 10H6); anti-bam (mouse, 1:10); anti-GFP (chicken, 1:10,000, Abcam 13970); anti-pH3 (rabbit, 1:100) ; anti-Hts (mouse, 1:10, 1B1); anti-cindr (rabbit, 1:200, a gift from Kaisa Haglund); anti-Fas3 (7G10; 1:10); TUNEL (In Situ Cell Death Detection Kit, TMR Red, Sigma/Roche); anti-Kmg (Fuller lab).

### Fluorescence microscopy and image analysis

Fluorescent images were taken with a Leica SP8 confocal. Laser intensity and detector gain for the confocal microscope or exposure time were adjusted for each experiment to ensure that the signal was in linear range and not saturated. Once image acquisition settings were determined, the same settings were maintained for the entire set of images be compared. Immunofluorescence images were processed using ImageJ with all samples to be compared going through the same processing.

### ASAP analysis of differentially expressed transcripts

ASAP was accessed at https://asap.epfl.ch/ and differential expression analysis was performed between cluster 62 (cyst stem cells) and 36 (early cyst cells) of Leiden resolution 6.0 of the testis snRNAseq dataset (Li et al., 2022) to identify transcripts upregulated at the earliest stages of cyst cell differentiation. The parameters used were the Wilcoxin [Seurat] method of differential expression, minimum % cells > 0.1, and fold change threshold of 1.3.

## Supplementary Material

Supplement 1

## Figures and Tables

**Table 1.** Results of targeted RNAi screen in cyst cells using c587Gal4 with temperature sensitive Gal80. Excel spreadsheet indicating testis phenotype for the 255 RNAi lines crossed to *c587Gal4, Gal80*^*ts*^ from male flies shifted to 30°C for 7 days. Includes information on gene name, ID, FBgn, log_2_FC from differential expression analysis of cluster 62 vs cluster 36, VDRC or Bloomington line number, whether a phenotype was observed, and description of phenotype observed.
