# Supplementary material for: Differentiating spermatogonia trigger somatic support cells to form septate junctions required for germ cell survival
Source: bioRxiv. 2026 Jun 8:2024.04.02.587826. Originally published 2024 Apr 3. Preprint. [Version 2] doi: 10.1101/2024.04.02.587826 (PMC11014526; doi:10.1101/2024.04.02.587826)

bioRxiv preprint doi: https://doi.org/10.1101/2024.04.02.587826; this version posted June 8, 2026. The copyright holder for this preprint (which was not certified by peer review) is the author/funder, who has granted bioRxiv a license to display the preprint in perpetuity. It is made available under a CC-BY 4.0 International license.

Supplemental Figure 1. Cyst cell RNAi screen of differentially expressed transcripts reveals novel genes required in cyst cells for differentiation of proliferating spermatogonia. (A-D) Phase-contrast images of the apical region of testes from male *c587Gal4, Gal80*^{ts} flies shifted to 30°C for 7 days with (A) no RNAi transgene (n=8) or an RNAi transgene targeting (B) *Ama* (n=8), (C) *Tre1* (n=9), or (D) *Pde9* (n=10). (E, F) Immunofluorescence images of the apical region of testes stained with (red) anti-Vasa and (green) anti-LolaF and (pink) anti-ph3 antibodies from male *c587Gal4, Gal80*^{ts} flies shifted to 30°C for 7 days that had (E) no RNAi transgene (n=10) or (F) an RNAi transgene targeting *Pde9* (n=10). (G, H) Immunofluorescence images stained with (red)

bioRxiv preprint doi: https://doi.org/10.1101/2024.04.02.587826; this version posted June 8, 2026. The copyright holder for this preprint (which was not certified by peer review) is the author/funder, who has granted bioRxiv a license to display the preprint in perpetuity. It is made available under a CC-BY 4.0 International license.

anti-Vasa, (green) anti-PH3 and (teal) anti-TJ from *c587Gal4, Gal80^{ts}* male flies shifted to 30°C for 7 days that had (G) no RNAi transgene (n=9) or a (H) *Pde9* RNAi transgene (n=12). Dotted outline: spermatogonia, curved bracket: spermatocytes, asterisk: hub; arrowhead: dividing spermatogonia near hub; arrows: dividing spermatogonia distal to hub. Scale bars: 50 μm.

Supplemental Figure 2. Knockdown of *side-V* in cyst cells triggers loss of germ cells at the 4-cell or early 8-cell stage. (A-D) Immunofluorescence images of the apical region of testes stained with (red) anti-Vasa, (teal) anti-TJ, and (yellow/white) fluorescently-labeled EdU incorporated into cells in S phase from male *c587Gal4, Gal80^{ts}* flies shifted to 30°C for 7 days that had (A) no RNAi transgene (n=13), (B) an RNAi transgene targeting *Nrx-IV* (n=11), or (C, D) an RNAi transgene targeting *side-V* (n=10). Scale bars: 50 μm.

Supplemental Figure 3. Cyst cells lacking *Nrx-IV* can differentiate, and cyst cells lacking *Nrx-IV* or *side-V* have increased JNK pathway activation. (A, B) Immunofluorescence images of the apical region of testes stained with (green) anti-Zfh1, (blue) anti-TJ, and (red) anti-Eya antibodies from male *c587Gal4, Gal80^{ts}* flies shifted to 30°C for 7 days that had (A) no RNAi transgene (n=6) or (B) an RNAi transgene targeting *Nrx-IV* (n=5). (C, D) Immunofluorescence images of the apical region of testes stained with (red) anti-Vasa and (green/white) anti-LacZ antibodies from male *c587Gal4, Gal80^{ts}* flies that had a *pucLacZ* transgene and an RNAi transgene targeting *Nrx-IV* shifted to 30°C for (C) 0 days as control (n=8) or (D) 7 days (n=8) to induce knockdown. (E, F) Immunofluorescence images of the apical region of testes stained with (red) anti-Vasa and (green/white) anti-LacZ antibodies from male

bioRxiv preprint doi: https://doi.org/10.1101/2024.04.02.587826; this version posted June 8, 2026. The copyright holder for this preprint (which was not certified by peer review) is the author/funder, who has granted bioRxiv a license to display the preprint in perpetuity. It is made available under a CC-BY 4.0 International license.

c587Gal4, Gal80^{ts} flies that had a *pucLacZ* transgene and were shifted to 30°C for 6 days to induce expression of (E) no RNAi transgene (n=5) or (F) an RNAi transgene targeting *side-V* (n=10). Arrows: differentiated cyst cell. Scale bars: 50 μm.

Table 1. Results of targeted RNAi screen in cyst cells using c587Gal4 with temperature sensitive Gal80. Excel spreadsheet indicating testis phenotype for the 255 RNAi lines crossed to *c587Gal4, Gal80^{ts}* from male flies shifted to 30°C for 7 days. Includes information on gene name, ID, FBgn, $\log_2$FC from differential expression analysis of cluster 62 vs cluster 36, VDRC or Bloomington line number, whether a phenotype was observed, and description of phenotype observed.

bioRxiv preprint doi: https://doi.org/10.1101/2024.04.02.587826; this version posted June 8, 2026. The copyright holder for this preprint (which was not certified by peer review) is the author/funder, who has granted bioRxiv a license to display the preprint in perpetuity. It is made available under a CC-BY 4.0 International license.

Supplemental Figure 1: Cyst cell RNAi screen of differentially expressed transcripts reveals novel genes required in cyst cells for differentiation of proliferating spermatogonia

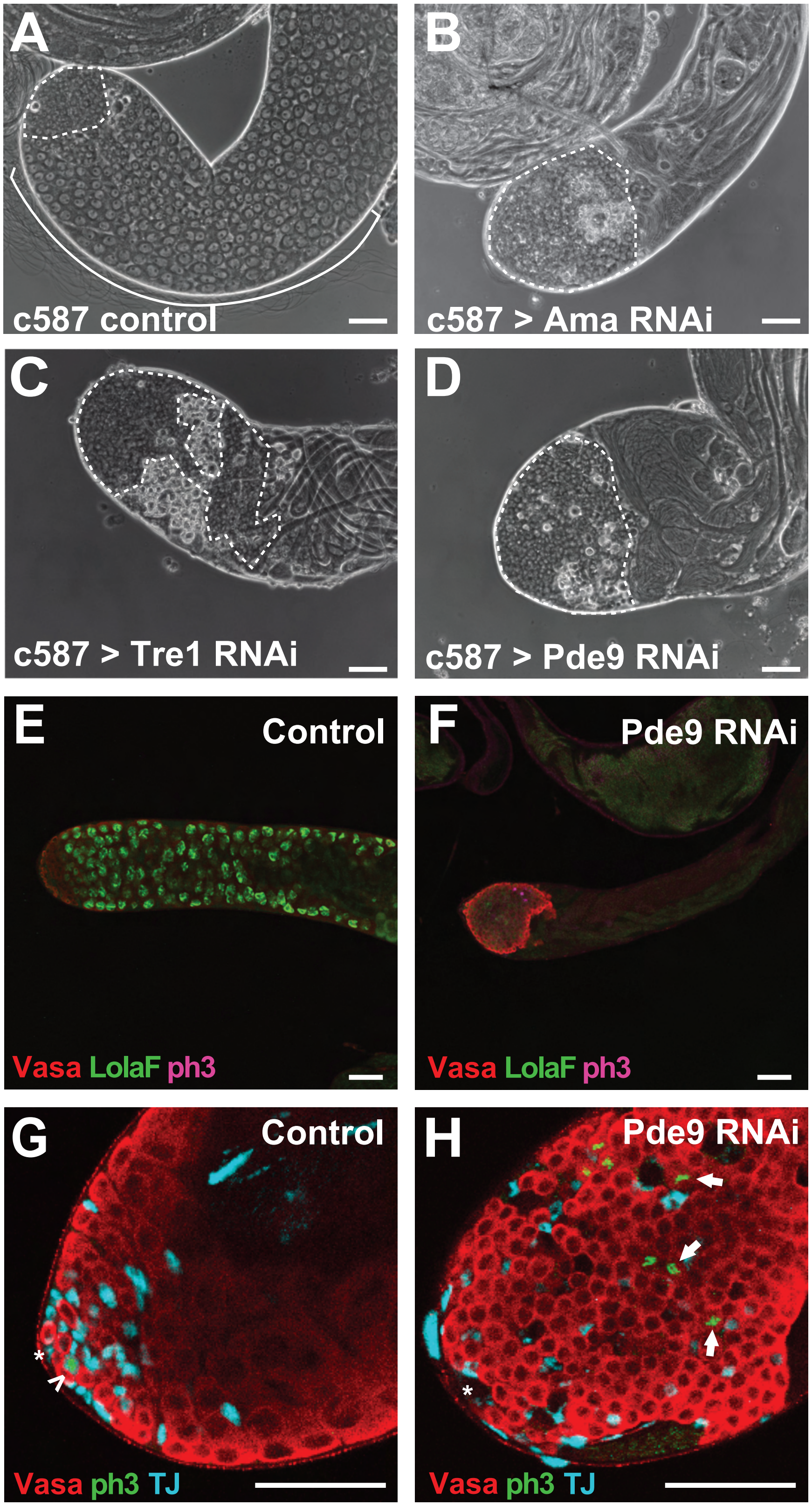

bioRxiv preprint doi: https://doi.org/10.1101/2024.04.02.587826; this version posted June 8, 2026. The copyright holder for this preprint (which was not certified by peer review) is the author/funder, who has granted bioRxiv a license to display the preprint in perpetuity. It is made available under a CC-BY 4.0 International license.

Supplemental Figure 2: Knockdown of side-V in cyst cells triggers loss of germ cells at the 4-cell or early 8-cell stage

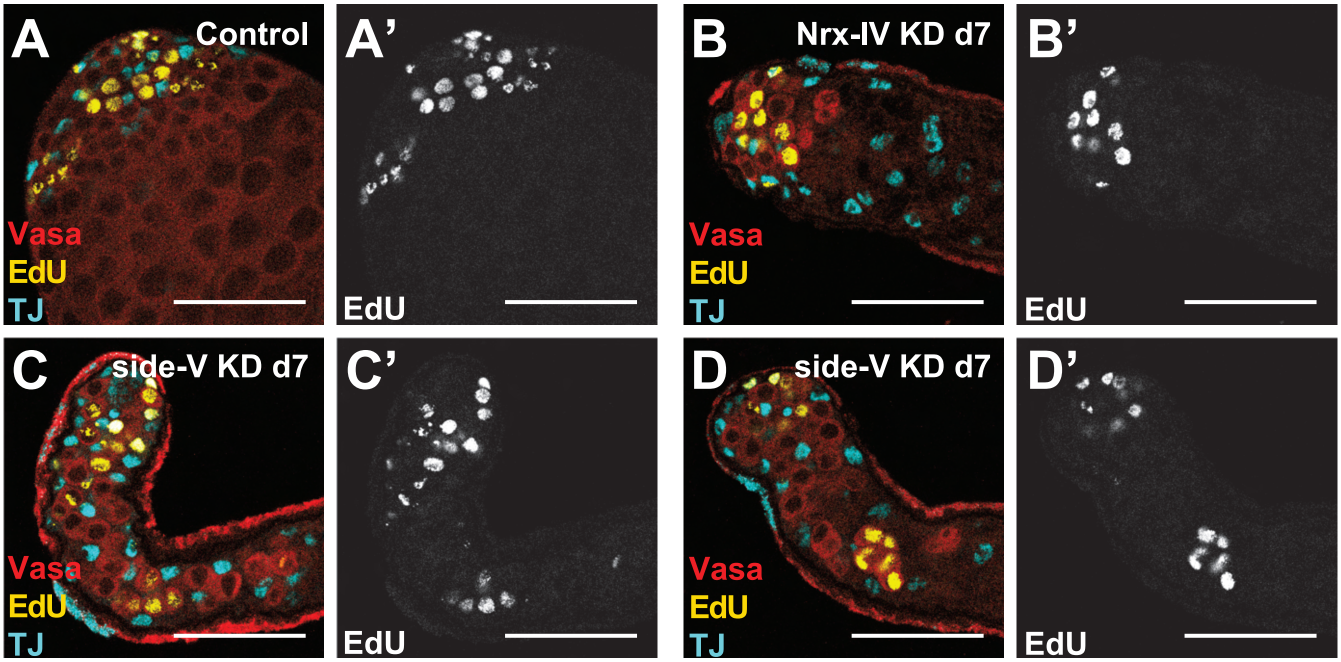

bioRxiv preprint doi: https://doi.org/10.1101/2024.04.02.587826; this version posted June 8, 2024. The copyright holder for this preprint (which was not certified by peer review) is the author/funder, who has granted bioRxiv a license to display the preprint in perpetuity. It is made available under a CC-BY 4.0 International license.

Supplemental Figure 3: Cyst cells lacking Nrx-IV can differentiate, but cyst cells lacking Nrx-IV or side-V have increased JNK pathway activation

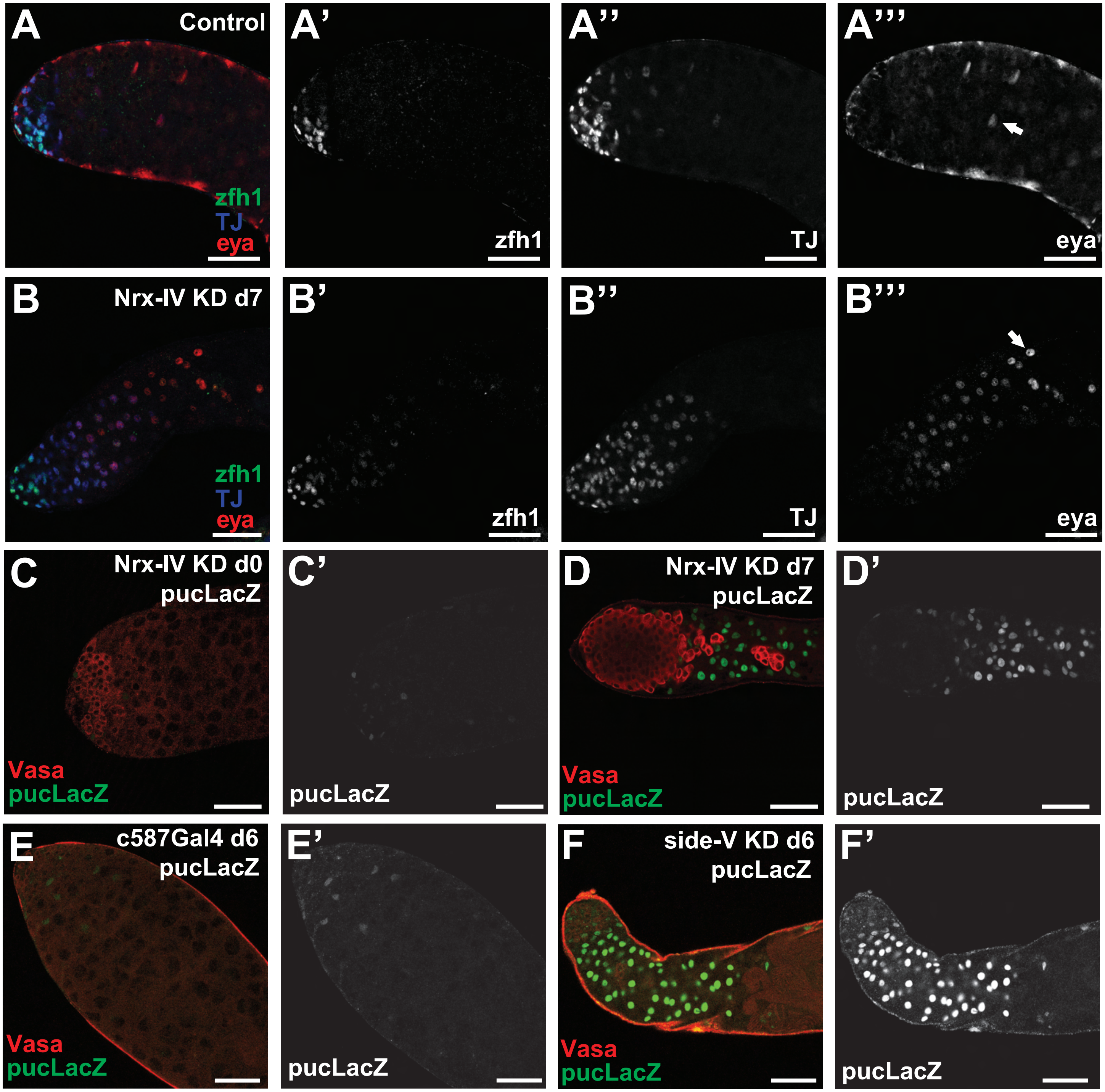

Supplement: Supplementary file 1 [file NIHPP2024.04.02.587826v2-supplement-2.pdf]
